# Two-dimensional biothermomechanical effects in a layer of skin tissue exposed to variable thermal loading using a fourth-order MGT model

**DOI:** 10.1038/s41598-025-01745-1

**Published:** 2025-05-16

**Authors:** Sami F. Megahid

**Affiliations:** 1 Department of Mathematics, Faculty of Science, Mansoura University, Mansoura, 35516 Egypt; 2 Department of Mathematics, Faculty of Science, New Mansoura University, New Mansoura City, 35712 Egypt

**Keywords:** Two-dimensional skin tissue, Fourth-order MGT thermoelastic model, Variable thermal loading, Normal mode method, Biological techniques, Mathematics and computing

## Abstract

A key consideration in medical procedures like thermal therapy is the danger of thermal harm to skin tissues from exposure to fluctuating thermal loads. To maximize treatment effectiveness while safeguarding healthy tissues, it is crucial to accurately anticipate and manage this damage, especially in hyperthermia therapy. The fourth-order Moore–Gibson–Thompson (4MGT) idea is employed in this study to lay a theoretical foundation for bioheat analysis. The purpose of this work is to clarify how skin tissues respond biothermally to varying thermal loading. The model developed makes it easier to anticipate the thermal reactions that occur in human skin and to estimate the efficiency of biothermal transfer in biological tissues. For the suggested model, a two-dimensional skin layer is utilized. The analytical results for tissue temperature are obtained using the normal mode approach. Both the impact of the duration of heat loading exposure and thermal damage are examined. Furthermore, the accuracy of the suggested model is evaluated by contrasting the obtained analytical results with accepted theories. The findings show that when the thermal relaxation time constant is included, the modified Moore-Gibson-Thomson biothermal model forecasts a decrease in temperature compared to the Pennes model.

## Introduction

Many thermal treatment methods are specifically designed to target injured skin tissues in the medical profession with the least amount of damage to surrounding undamaged tissue. Heat ablation has shown great promise in the medical field for laser procedures, freckle removal, wound healing, and cancer treatment. There are several methods for generating heat when it comes to thermal ablation^1^. RF waves, infrared radiation, microwave radiation, thermoseeds with magnetic excitability, and other heat emitters are among them. It’s crucial to keep in mind, nevertheless, that high temperatures can damage healthy cells because they can cause denaturation of proteins and rupture of cellular membranes^2^. The greatest possible therapeutic outcomes and patient health depend on the careful regulation of temperature gradients inside the tissues. The projected temperature distribution must be known before starting thermal treatments because it is challenging to monitor comprehensive real-time temperature charts. More research is required to advance our understanding of how humans’ bodies respond to hyperthermia.

The heat effects of blood in biological tissues, including perfusion of blood and convection, should be considered to accurately characterize the mechanisms of thermal transfer in living things, especially with regard to heat therapeutic benefits. Physiological processes also need to be considered, especially the production of metabolic heat. First introduced by Pennes in 1948, the Pennes bioheat equation is a widely used mathematical model for studying heat transport in biological tissues, particularly skin^3^. It extends the standard heat equation by incorporating blood circulation and metabolic heat generation^4^. Numerous heat transport mechanisms in tissues are taken into account by the model, including conduction, metabolic heat production, and blood perfusion. The Pennes bioheat equation is used in many different contexts, such as thermal ablation, cryotherapy, and hyperthermia, to predict temperature distribution and potential thermal damage within tissues. However, the model has limitations, including neglecting the pulsatility and nonuniformity of blood flow and assuming constant temperature characteristics. Several changes and modifications have been proposed to improve the accuracy and applicability of the Pennes bioheat equation in various biological systems^5^. One important use of the Pennes bioheat equation is thermal therapy, which employs heat to kill malignant cells or tissues. The equation facilitates the optimization of treatment parameters and minimizes side effects by forecasting the temperature distribution within the tissue during treatment^6^. Additionally, it can be utilized to predict heat damage in the tissue, which aids in assessing the efficacy of a treatment.

Fu et al.‘s primary focus was on how tumor form affected the distribution of skin temperature during thermal therapy^7^. They used the Pennes model to evaluate the heat response in this scenario. Similar to this, Singh and Repaka^8^ investigated the effects of radiofrequency ablation on the thermal characteristics of breast tumors using a multilayered breast model that includes the effects of temperature-dependent components like electrical and thermal conductivities. Zhang et al.^9^ employed the Pennes bioheat transfer model in steady-state conditions to investigate the effect of skin tissue temperature on blood perfusion rate. Temperature was found to have a linear, quadratic, and exponential association with perfusion. The model was solved using the boundary reciprocity approach.

Despite being widely utilized for a wide range of technical and thermal challenges, the standard Fourier heat conduction model has limitations in several situations. It’s interesting to note that experimental research has demonstrated that the Pennes bioheat transfer (parabolic) model can yield inaccurate results, particularly in situations with significant heat flow or extremely brief intervals^10^. This discrepancy arises from the Penne model’s inability to take tissue thermal inertia into consideration, which can lead to serious errors in temperature prediction. The thermal wave model is predicated on the premise that the temperature gradient and energy transfer happen quickly, which may not always be the case, even if it can accurately replicate biological materials^11^. Because biological tissues are naturally complex and non-uniform, there is a temporal delay in both the temperature gradient and energy transfer as well as the heat flux and temperature gradient. In order to account for thermal inertia, Cattaneo and Vernotte^12,13^ created the concept of thermal relaxation time. This number illustrates the time lag between the heat flux vector and the corresponding temperature gradient, particularly in processes requiring rapid heat transfer. Liu et al.^14^ acknowledged this limitation by proposing a novel bioheat transport model that uses a thermal wave approach. By incorporating the thermal relaxation period into the Pennes equation, this enhanced model offers a more accurate representation of unstable thermal transmission mechanisms in living organisms or in laboratory environments.

The thermal wave model accurately predicts the time response of heat transport on a small scale. However, it has a limited capacity to catch minute spatial thermal exchanges. Tzou et al.^15^ created the dual-phase lag (DPL) model to address this problem and investigate rapid heat transport in nonuniform materials. This model incorporates phase delays due to thermal inertia and microstructural interactions. The thermal wave bio-heat transfer and DPL models were then used to analyze heat transfer processes in biological tissues, taking into account heat sources including lasers^16–18^, microwaves^19^, and ultrasound^20^.

Generalized thermoelasticity theories, which assume a finite speed for thermal signals, have been of considerable interest for over 40 years^21–24^. These theories employ a hyperbolic heat equation in contrast to the standard coupled thermoelasticity (CT) theory, which is predicated on a parabolic heat equation and infinite heat propagation speed. By substituting a new heat transfer model for the standard Fourier law, Lord and Shulman (LS)^25^ were the first to generalize for isotropic materials, enabling the creation of a wave-type heat equation. A second generalization, the thermoelasticity theory (GL), which incorporates the rate of temperature change, was then presented by Green and Lindsay (GL)^26^. Green and Naghdi (GN)^27^ extended the theoretical framework with the GN theory, which deviates from previous models by omitting thermal energy dissipation.

The Moore–Gibson–Thompson (MGT) equation has attracted a lot of research interest because of its practical applicability in a range of fields, including industry and healthcare^27^. High-intensity ultrasound, which has numerous applications in lithotripsy, thermal therapy, and ultrasonic cleaning, has also been the subject of numerous investigations^27^. In lithotripsy, the temperature distribution that emerges from simulating the passage of ultrasonic waves through the body can be predicted using the MGT equation and other generalized thermoelasticity theories^28,29^. Quintanilla^30,31^ developed a novel approach to heat transmission in thermoelasticity by incorporating the MGT equation into the thermal conductivity equation. This model adds a relaxation component to the structure of the third-kind Green–Naghdi model^32^. Building on Quintanilla’s work, the references^33–35^ examined several new thermoelastic systems and their applications. The thermomechanical response to various engineering challenges and the complexities of fluid dynamics have also been studied using the modified model^36–38^.

The main goal of this work is to develop a more thorough and improved framework for thermoelasticity that better represents the relationship between temperature and elasticity than earlier models. This new model combines the Moore-Gibson-Thompson (MGT) equation with the Lord-Shulman and Green-Naghdi type III (GN-III) thermoelastic theories. Higher-order temporal derivatives of heat flow are incorporated into the updated model to accurately account for the intricate reactions of skin tissues to heating.

One of the study’s main accomplishments is the model’s superiority over previous generalized thermoelasticity models due to its ability to effectively depict the behavior of skin tissues. Furthermore, the suggested paradigm provides improved predictions and insights into the thermoelastic response of skin tissues by clarifying the complex link between temperature and elasticity.

Skin tissues subjected to varying heat stress are directly modeled using the fourth-order MGT thermal model. The analytical results for tissue are obtained using the normal mode approach. The behavior of the system is examined using a range of methodologies, including graphical and analytical approaches. The paper discusses important topics like the frequency of angular thermal loading and the consequences of thermal relaxation in addition to thermal damage. When compared to experimental data, the results demonstrate that the proposed model is very accurate, emphasizing its ability to improve healthcare planning and execution.

## Mathematical equations

Heat transport in skin tissues is governed by the well-known and widely used Fourier’s law of heat transfer. According to Fourier’s law, a material’s internal temperature gradient controls how quickly heat passes through it. The following equation represents this association:

1 $$\:\overrightarrow{h}\left(\overrightarrow{p},\:t\right)=-{\mathcal{K}}_{t}\nabla\:\theta\:\left(\overrightarrow{p},\:t\right).$$

In this equation, $\:{\mathcal{K}}_{t}$ stands for the skin tissue’s thermal conductivity, whereas $\:\overrightarrow{h}$ indicates the heat flux at a certain point in time $\:t$ and place $\:\overrightarrow{p}$. The variable $\:\theta\:=T-{T}_{b}$ represents the temperature differential between arterial blood ($\:{T}_{b}$) and tissue ($\:T$).

Within living biological tissues, heat is transferred by three primary mechanisms: heat conduction, convective heat transfer, and blood perfusion. Fourier’s law of heat conduction states that when thermal energy is transferred through a material due to a temperature gradient, heat conduction occurs inside the tissue. The blood’s movement promotes convective heat transmission between the blood and the surrounding tissue, which in turn facilitates thermal energy exchange. Among the factors influencing this process are the blood vessel surface area, the temperature differential between the blood and tissue, and the blood flow rate. Blood perfusion, or the flow of blood through capillaries and other blood vessels within the tissue, also has a significant effect on the entire process of heat transmission. The interaction of blood flow with surrounding tissue affects the complex thermal dynamics observed in biological systems.

Among the models developed to answer Fourier’s law are those put forth by Green and Naghdi^27,32^. Some of the models that have been developed are these. There are several ways to display the augmented Fourier law from the GN-III model, including

2 $$\:\overrightarrow{h}=-{\mathcal{K}}_{t}\nabla\:\theta\:-{\mathcal{K}}_{t}^{*}\nabla\:\varnothing\:,$$

with $\:{\mathcal{K}}_{t}^{*}$ representing the rate of thermal conductivity. $\:\nabla\:\varnothing\:$ is regarded as a novel constitutive variable in Green and Naghdi’s ideas. Additionally, $\:\varnothing\:$ is read as the thermal-displacement function, which is the equivalent of the mechanical displacement in mechanical fields. $\:\frac{\partial\:\varnothing\:}{\partial\:t}=\theta\:$ is satisfied by the thermal displacement function.

Quintanilla^31^ created a new version of the GN-III heat conduction model inside the context of the MGT equation by adding the relaxation parameter $\:{\tau\:}_{h}$. The updated Fourier formula was as follows:

3 $$\:\left(1+{\tau\:}_{h}\frac{\partial\:}{\partial\:t}+\frac{1}{2}{{\tau\:}_{h}}^{2}\frac{{\partial\:}^{2}}{\partial\:{t}^{2}}\right)\overrightarrow{h}=-{\mathcal{K}}_{t}\nabla\:\theta\:-{\mathcal{K}}_{t}^{*}\nabla\:\varnothing\:.$$

The MGT model is based on the following: Viscoelastic Behavior: The material possesses both viscous and elastic qualities, which means that both the rate of deformation and the existing deformation determine how the material reacts to stress or strain and Nonlocal Effects: The model takes into account nonlocal interactions, which means that a point’s behavior is dependent on both the local conditions and the conditions in the surrounding area. The modeling of distributed interactions and effects, like dispersive waves, is made possible by this incorporation.

Since it describes the connection between temperature, the heat transfer coefficient, and energy flow, the energy balance equation for heat transfer is among the most crucial basic equations that may be applied in the field of heat transfer analysis. The phrase for this is

4 $$\:{\rho\:}_{t}{c}_{t}\frac{\partial\:\theta\:}{\partial\:t}+{T}_{b}{\gamma\:}_{t}\frac{\partial\:e}{\partial\:t}+\nabla\:\cdot\:\overrightarrow{h}={S}_{t},$$

where $\:{\gamma\:}_{t}$ is the thermal modulus, which is determined using the formula $\:{\gamma\:}_{t}={\alpha\:}_{t}\left(3{\lambda\:}_{t}+2{\mu\:}_{t}\right)$, $\:{c}_{t}$ is the tissue specific heat, and $\:{\rho\:}_{t}$ is the tissue density. In this case, the character $\:{\alpha\:}_{t}$ stands for the thermal expansion coefficient, whereas the symbols $\:{\mu\:}_{t}$ and $\:{\lambda\:}_{t}$ represent Lame’s constants. The symbol for the cubical dilatation, $\:e$, is

5 $$\:e={u}_{i,i}=\text{div}\overrightarrow{\text{u}},$$

where $\:{u}_{i}$ stands for each of the displacement’s constituent parts. $\:{S}_{t}$ takes into consideration both external and interior heat sources, as indicated by^39,40^

6 $$\:{S}_{t}={S}_{b}+{S}_{met}+{S}_{ext}.$$

The amount of heat produced by tissue metabolism is represented by $\:{S}_{met}$, the heat energy from an external source by $\:{S}_{ext}$, and the heat source from blood circulation by $\:{S}_{b}.$

After finishing the time derivation process for Eqs. (3) and (4) and combining them, the fourth-order MGT thermal conductivity equation can be produced as follows:

7 $$\:\begin{array}{c}\left[1+{\tau\:}_{h}\frac{\partial\:}{\partial\:t}+\frac{1}{2}{{\tau\:}_{h}}^{2}\frac{{\partial\:}^{2}}{\partial\:{t}^{2}}\right]\left[{\rho\:}_{t}{c}_{t}\frac{{\partial\:}^{2}\theta\:}{\partial\:{t}^{2}}\:+{T}_{b}{\gamma\:}_{t}\frac{{\partial\:}^{2}e}{\partial\:{t}^{2}}-\frac{\partial\:}{\partial\:t}\left({S}_{b}+{S}_{met}+{S}_{ext}\right)\right]\\\:\:\:\:\:\:\:\:\:\:\:\:\:\:\:\:\:\:\:\:\:\:\:\:\:\:\:\:\:\:\:\:\:\:\:\:\:\:\:\:\:\:\:\:\:\:\:\:\:\:\:\:={\mathcal{K}}_{t}{\nabla\:}^{2}\frac{\partial\:\theta\:}{\partial\:t}+{\mathcal{K}}_{t}^{*}{\nabla\:}^{2}\theta\:.\end{array}$$

In addition to the basic equation of thermoelasticity, equations of motion and constitutive relationships are crucial components when developing thermoelastic models. The following formula applies to the general versions of these equations^41,42^

8 $$\:{\rho\:}_{t}\frac{{\partial\:}^{2}{u}_{i}}{\partial\:{t}^{2}}={\lambda\:}_{t}{u}_{j,ij}+{\mu\:}_{t}\left({u}_{j,ij}+\:{u}_{i,jj}\right)+{\rho\:}_{t}{F}_{i}-{\gamma\:}_{t}{\theta\:}_{,i},$$

9 $$\:{\sigma\:}_{ij}=2{\mu\:}_{t}{e}_{ij}+{\delta\:}_{ij}\left[{\lambda\:}_{t}{e}_{kk}-{\gamma\:}_{t}\theta\:\right],$$

10 $$\:2{e}_{ij}={u}_{i,j}+{u}_{j,i}.$$

The symbol $\:{\sigma\:}_{ij}$ represents the stress tensor in the given framework, while the strain tensor is represented by the notation $\:{e}_{ij}$. The symbol $\:{F}_{i}$ represents the vector components of the external body force.The Kronecker delta function is represented by the symbol $\:{\delta\:}_{ij}.$

### Mathematical formulation for the problem

**Fig. 1:**
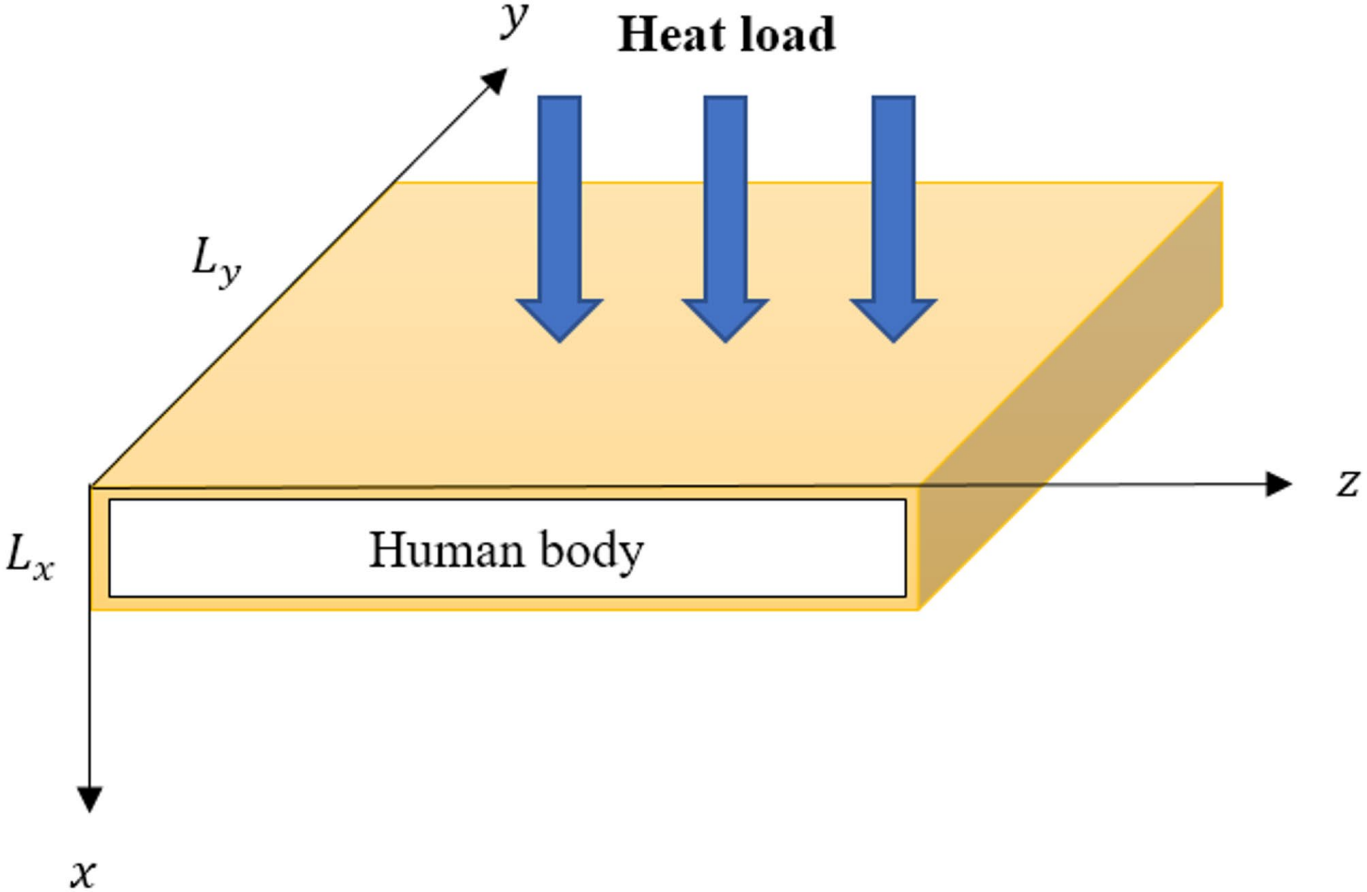
Schematic diagram of skin tissue exposed to heat load.

The case of a 2D skin tissue in the interval $\:0\le\:x\le\:{L}_{x}$ and $\:0\le\:y\le\:{L}_{y}$ will be covered in this section (see Fig. 1). Cartesian coordinates $\:(x,\:y,\:z)$ will be used in order to meet the needs of the challenge. In addition to the time variable $\:t$, the domain variables under study will be functions of $\:x$ and $\:y$ coordinates because the problem is 2D. The plane $\:x=0$ will serve as the free surface, and the $\:x$ -axis will be plumb downward into the medium’s depth.

By considering both thermal and mechanical boundary conditions, the proposed problem was resolved. The skin tissue’s outside surface was believed to be heated in a time-dependent way and to be traction-free, whereas the inner surface was believed to be traction-free and insensitive to temperature changes. The following boundary conditions are therefore made possible:

11 $$\:\begin{array}{c}\theta\:\left(x,\:y,t\right)=h\left(y,\:t\right),\:{\sigma\:}_{xx}\left(x,\:y,\:t\right)={\sigma\:}_{xy}\left(x,\:y,\:t\right)=0\:\:\:at\:\:\:x=0,\\\:\theta\:\left(x,\:y,t\right)={\sigma\:}_{xx}\left(x,\:y,\:t\right)={\sigma\:}_{xy}\left(x,\:y,\:t\right)=0\:\:\:at\:\:\:x={L}_{x},\end{array}$$

where the heat load applied to the skin tissue’s outer surface is denoted by $\:h\left(y,\:t\right).$

The displacement components have the following form

12 $$\:{u}_{x}={u}_{x}\left(x,\:y,\:t\right),\:\:{u}_{y}={u}_{y}\left(x,\:y,\:t\right),\:\:{u}_{z}=0.$$

Equations (10) and (12) can be used to derive the strain components listed below

13 $$\:{e}_{xx}=\frac{\partial\:{u}_{x}}{\partial\:x},\:\:{e}_{yy}=\frac{\partial\:{u}_{y}}{\partial\:y},\:\:{e}_{xy}=\frac{1}{2}\left(\frac{\partial\:{u}_{x}}{\partial\:y}+\frac{\partial\:{u}_{y}}{\partial\:x}\right),\:\:{e}_{xz}={e}_{yz}={e}_{zz}=0.$$

It is possible to obtain the stress components by applying Eqs. (9) and (13) as

14 $$\:{\sigma\:}_{xx}=\left({\lambda\:}_{t}+2{\mu\:}_{t}\right)\frac{\partial\:{u}_{x}}{\partial\:x}+{\lambda\:}_{t}\frac{\partial\:{u}_{y}}{\partial\:y}-{\gamma\:}_{t}\theta\:,$$

15 $$\:{\sigma\:}_{yy}=\left({\lambda\:}_{t}+2{\mu\:}_{t}\right)\frac{\partial\:{u}_{y}}{\partial\:y}+{\lambda\:}_{t}\frac{\partial\:{u}_{x}}{\partial\:x}-{\gamma\:}_{t}\theta\:,$$

16 $$\:{\sigma\:}_{xy}={\mu\:}_{t}\left(\frac{\partial\:{u}_{x}}{\partial\:y}+\frac{\partial\:{u}_{y}}{\partial\:x}\right).$$

Equations (5) and (12) can be substituted into Eq. (8) to produce the following

17 $$\:\left({\lambda\:}_{t}+{\mu\:}_{t}\right)\frac{\partial\:e}{\partial\:x}+{\mu\:}_{t}{\nabla\:}^{2}{u}_{x}-{\gamma\:}_{t}\frac{\partial\:\theta\:}{\partial\:x}={\rho\:}_{t}\frac{{\partial\:}^{2}{u}_{x}}{\partial\:{t}^{2}},$$

18 $$\:\left({\lambda\:}_{t}+{\mu\:}_{t}\right)\frac{\partial\:e}{\partial\:y}+{\mu\:}_{t}{\nabla\:}^{2}{u}_{y}-{\gamma\:}_{t}\frac{\partial\:\theta\:}{\partial\:y}={\rho\:}_{t}\frac{{\partial\:}^{2}{u}_{y}}{\partial\:{t}^{2}}.$$

The current analysis makes use of the following blood perfusion term^43^

19 $$\:{S}_{b}=-{w}_{b}{\rho\:}_{b}{c}_{b}\theta\:.$$

The heat exchange between the tissue and the blood flow is described by this phrase, where $\:{w}_{b}$ is the rate of blood perfusion, $\:{\rho\:}_{b}$ is the blood mass density, and $\:{c}_{b}$ is the blood specific heat.

The temperature of the tissue has an impact on the body’s metabolic heat generation $\:{S}_{met}$, especially for skin tissue that contains blood vessels. The metabolic heat source $\:{S}_{met}$ is represented as follows^44^

20 $$\:{S}_{met}={S}_{im}(1+{c}_{m}\theta\:),$$

where $\:{S}_{im}$ is the rate at which heat is generated by the baseline metabolic process, and $\:{c}_{m}$ is the temperature coefficient that influences the rate of metabolic heat synthesis.

The heat conduction Eq. (7) can be written as follows when the heat energy coming from an external source is absent ($\:{S}_{ext}=0$):

21 $$\:\begin{array}{c}\left[1+{\tau\:}_{h}\frac{\partial\:}{\partial\:t}+\frac{1}{2}{{\tau\:}_{h}}^{2}\frac{{\partial\:}^{2}}{\partial\:{t}^{2}}\right]\left[{\rho\:}_{t}{c}_{t}\frac{{\partial\:}^{2}\theta\:}{\partial\:{t}^{2}}\:+{T}_{b}{\gamma\:}_{t}\frac{{\partial\:}^{2}e}{\partial\:{t}^{2}}-({c}_{m}{S}_{im}-{w}_{b}{\rho\:}_{b}{c}_{b})\frac{\partial\:\theta\:}{\partial\:t}\right]\\\:\:\:\:\:\:\:\:\:\:\:\:\:\:\:\:\:\:\:\:\:\:\:\:\:\:\:\:\:\:\:\:\:\:\:\:\:\:\:\:\:\:\:\:\:\:\:\:\:\:\:\:=\left({\mathcal{K}}_{t}\frac{\partial\:}{\partial\:t}+{\mathcal{K}}_{t}^{*}\right){\nabla\:}^{2}\theta\:.\end{array}$$

The addition of dimensionless variables simplifies the problem’s governing mathematical equations. Equations (17), (18), and (21) are made dimensionless by adding the following dimensionless values

22 $$\:\begin{array}{c}\left\{{\:x}^{{\prime\:}},\:{y}^{{\prime\:}},\:{{u}_{x}}^{{\prime\:}},{{u}_{y}}^{{\prime\:}}\right\}=\mathcal{c}\zeta\:\left\{x,\:y,\:{u}_{x},\:{u}_{y}\right\},\:\left\{{t}^{{\prime\:}},\:{{\tau\:}_{h}}^{{\prime\:}}\right\}={\mathcal{c}}^{2}\zeta\:\left\{t,{\tau\:}_{h}\right\},\:\:\\\:\:\:{\theta\:}^{{\prime\:}}=\frac{{\gamma\:}_{t}\theta\:}{{\lambda\:}_{t}+2\:{\mu\:}_{t}},\:\:\:{{\sigma\:}_{ij}}^{{\prime\:}}=\frac{{\sigma\:}_{ij}}{{\mu\:}_{t}},\:\:\:{\mathcal{c}}^{2}=\frac{{\lambda\:}_{t}+2\:{\mu\:}_{t}}{{\rho\:}_{t}},\:\:\:\zeta\:=\frac{{\rho\:}_{t}{c}_{t}}{{\mathcal{K}}_{t}}.\end{array}$$

By substituting the dimensionless variables from Eq. (31) into Eqs. (17), (18), and (21), we reduce the fundamental equations as follows (It removes the prime notation)

23 $$\:\left({\beta\:}^{2}-1\right)\frac{\partial\:e}{\partial\:x}+{\nabla\:}^{2}{u}_{x}{-\beta\:}^{2}\frac{\partial\:\theta\:}{\partial\:x}={\beta\:}^{2}\frac{{\partial\:}^{2}{u}_{x}}{\partial\:{t}^{2}},$$

24 $$\:\left({\beta\:}^{2}-1\right)\frac{\partial\:e}{\partial\:y}+{\nabla\:}^{2}{u}_{y}{-\beta\:}^{2}\frac{\partial\:\theta\:}{\partial\:y}={\beta\:}^{2}\frac{{\partial\:}^{2}{u}_{y}}{\partial\:{t}^{2}},$$

25 $$\:\left(\epsilon\:+\frac{\partial\:}{\partial\:t}\right){\nabla\:}^{2}\theta\:=\left(1+{\tau\:}_{h}\frac{\partial\:}{\partial\:t}+\frac{1}{2}{{\tau\:}_{h}}^{2}\frac{{\partial\:}^{2}}{\partial\:{t}^{2}}\right)\left[\frac{{\partial\:}^{2}\theta\:}{\partial\:{t}^{2}}-{\epsilon\:}_{1}\frac{\partial\:\theta\:}{\partial\:t}+{\epsilon\:}_{2}\frac{{\partial\:}^{2}e}{\partial\:{t}^{2}}\right],$$

where

26 $$\:{\beta\:}^{2}=\frac{{\lambda\:}_{t}+2{\mu\:}_{t}}{{\mu\:}_{t}},\:\epsilon\:=\frac{{\mathcal{K}}_{t}^{*}}{{\mathcal{K}}_{t}\zeta\:{\mathcal{c}}^{2}},\:{\epsilon\:}_{1}=\frac{{w}_{b}{\rho\:}_{b}{c}_{b}-{S}_{im}{c}_{m}}{{\mathcal{c}}^{2}{\zeta\:}^{2}{\mathcal{K}}_{t}},\:{\epsilon\:}_{2}=\frac{{{\gamma\:}_{t}}^{2}{T}_{b}}{{\rho\:}_{t}{c}_{t}({\lambda\:}_{t}+2{\mu\:}_{t})}.$$

The constitutive formulas diminish to

27 $$\:{\sigma\:}_{xx}=\left({\beta\:}^{2}-2\right)e+2\frac{\partial\:{u}_{x}}{\partial\:x}-{\beta\:}^{2}\:\theta\:,$$

28 $$\:{\sigma\:}_{yy}=\left({\beta\:}^{2}-2\right)e+2\frac{\partial\:{u}_{y}}{\partial\:y}{-\beta\:}^{2}\:\theta\:,$$

29 $$\:{\sigma\:}_{xy}=\frac{\partial\:{u}_{x}}{\partial\:y}+\frac{\partial\:{u}_{y}}{\partial\:x}.$$

After being differentiated with regard to x and y, respectively, Eqs. (33) and (34) are added

30 $$\:{(\nabla\:}^{2}-\frac{{\partial\:}^{2}}{\partial\:{t}^{2}})e-{\nabla\:}^{2}\:\theta\:=0.$$

#### Normal mode solution and damage prediction

The following type of normal mode can be used to break down the solution of the many variables being studied

31 $$\:\left(\theta\:,\:{u}_{x},\:{u}_{y},\:{\sigma\:}_{ij},e\right)=\left(\stackrel{-}{\theta\:},\stackrel{-}{{u}_{x}},\stackrel{-}{{u}_{y}},\stackrel{-}{{\sigma\:}_{ij}},\stackrel{-}{e}\right)\left(x\right)\text{exp}\left(\text{i}wy+ft\right),$$

where $\:f$ is the complex frequency constant, $\:i$ is an imaginary unit, and $\:w$ is the wave number in the $\:y$ -direction. Equation (31), when inserted into Eqs. (25) and (30), yields

32 $$\:\begin{array}{c}\left[\left(f+\epsilon\:\right)({D}^{2}-{w}^{2})-\left(1+{\tau\:}_{h}\:f+\frac{1}{2}{{\tau\:}_{h}}^{2}{f}^{2}\right)f(f-{\epsilon\:}_{1})\right]\stackrel{-}{\theta\:}\left(x\right)=\\\:\left(1+{\tau\:}_{h}\:f+\frac{1}{2}{{\tau\:}_{h}}^{2}{f}^{2}\right){\epsilon\:}_{2}\:{f}^{2}\stackrel{-}{e}\left(x\right),\end{array}$$

33 $$\:{(D}^{2}-{w}^{2}-\:{f}^{2}\left)\:\stackrel{-}{e}\right(x){-(D}^{2}-{w}^{2})\:\stackrel{-}{\theta\:}\left(x\right)=0,$$

where $$\:D=\frac{d}{dx}.$$

The differential equation below is obtained by taking the variables $\:\stackrel{-}{\theta\:}\left(x\right)$ or $\:\stackrel{-}{e}\left(x\right)$ out of Eqs. (32) and (33)

34 $$\:\left({D}^{4}-A{D}^{2}+B\right)\{\stackrel{-}{e}\left(x\right),\:\stackrel{-}{\theta\:}\left(x\right)\}=0,$$

where

35 $$\begin{array}{*{20}c} {A = 2w^{2} + A_{1} ,~B = w^{4} + B_{1} w^{2} + B_{2} ,} \\ {A_{1} = f^{2} + \in \left( {f - \varepsilon _{1} + \varepsilon _{2} } \right),~B_{1} = f^{2} + \in \left( {f - \varepsilon _{1} + f\varepsilon _{2} } \right),} \\ {B_{2} = \in f^{2} \left( {f - \varepsilon _{1} } \right),~ = \frac{{f\left( {1 + \tau _{h} {\text{~}}f + \frac{1}{2}\tau _{h} ^{2} f^{2} } \right)}}{{f + \varepsilon }}.} \\ \end{array}$$

Equation (34) can be factored as follows

36 $$\:\left({D}^{2}-{k}_{1}^{2}\right)\left({D}^{2}-{k}_{2}^{2}\right)\{\stackrel{-}{e}\left(x\right),\:\stackrel{-}{\theta\:}\left(x\right)\}=0,$$

where $\:{k}_{j}^{2}\:(j=1,\:2)$ is the following characteristic equation’s root

37 $$\:{k}^{4}-A{k}^{2}+B=0.$$

Therefore, Eq. (34)’s solution is provided by

38 $$\:\left\{\stackrel{-}{e}\left(x\right),\:\stackrel{-}{\theta\:}\left(x\right)\right\}={\sum\:}_{n=1}^{2}\left[\left\{{R}_{n},\:{M}_{n}\right\}{\:\text{e}}^{{k}_{n}x}+\left\{{R}_{n+2},\:{M}_{n+2}\right\}{\:\text{e}}^{-{k}_{n}x}\right],$$

where the parameters $\:{R}_{l}$ and $\:{M}_{l}$ ($\:l=1,\:2,\:3,\:4$) depend on $\:w$ and $\:f.$

The following relationship is found when Eq. (38) is replaced into Eq. (33)

39 $$\:\left\{{M}_{n},\:{M}_{n+2}\right\}=\frac{{k}_{n}^{2}-{w}^{2}-{f}^{2}}{{k}_{n}^{2}-{w}^{2}}\left\{{R}_{n},\:{R}_{n+2}\right\}.$$

Upon substituting Eq. (39) into Eq. (38), we obtain

40 $$\:\left\{\stackrel{-}{e}\left(x\right),\:\stackrel{-}{\theta\:}\left(x\right)\right\}={\sum\:}_{n=1}^{2}\left\{1,\:\frac{{k}_{n}^{2}-{w}^{2}-{f}^{2}}{{k}_{n}^{2}-{w}^{2}}\right\}\left[{R}_{n}{\:\text{e}}^{{k}_{n}x}+{R}_{n+2}{\:\text{e}}^{-{k}_{n}x}\right].$$

The displacement $\:{u}_{x}$ is found by inserting Eqs. (31) and (40) into Eqs. (23) to get

41 $$\:{(D}^{2}-{m}^{2}\left)\:\stackrel{-}{{u}_{x}}\right(x)={\sum\:}_{n=1}^{2}\frac{{k}_{n}\left({k}_{n}^{2}-{w}^{2}-{\beta\:}^{2}{f}^{2}\right)}{{k}_{n}^{2}-{w}^{2}}\left[{R}_{n}{\:\text{e}}^{{k}_{n}x}-{R}_{n+2}{\:\text{e}}^{-{k}_{n}x}\right],$$

where

42 $$\:{m}^{2}={w}^{2}+\:{\beta\:}^{2}{f}^{2}.$$

The solution to Eq. (41) is provided by

43 $$\:\stackrel{-}{{u}_{x}}\left(x\right)={N}_{1}{w}^{2}{\text{e}}^{mx}+{N}_{2}{w}^{2}{\text{e}}^{-mx}+{\sum\:}_{n=1}^{2}\frac{{k}_{n}}{{k}_{n}^{2}-{w}^{2}}[{R}_{n}{\:\text{e}}^{{k}_{n}x}-{R}_{n+2}{\:\text{e}}^{-{k}_{n}x}],$$

where the parameters $\:{N}_{1}$ and $\:{N}_{2}$ are dependent on $\:w$ and $\:f.$

Using the cubical dilatation and Eq. (31), we can determine that

44 $$\:\stackrel{-}{{u}_{y}}\left(x\right)=-\frac{i}{w}\left(\stackrel{-}{e}\left(x\right)-\frac{d\stackrel{-}{{u}_{x}}\left(x\right)}{dx}\right).$$

Equations (40) and (43) may be swapped into Eq. (44) to yield

45 $$\:\stackrel{-}{{u}_{y}}\left(x\right)=iw\left\{{N}_{1}m{\text{e}}^{mx}-{N}_{2}m{\text{e}}^{-mx}+{\sum\:}_{n=1}^{2}\frac{1}{{k}_{n}^{2}-{w}^{2}}\left[{R}_{n}{\:\text{e}}^{{k}_{n}x}+{R}_{n+2}{\:\text{e}}^{-{k}_{n}x}\right]\right\}.$$

The stress components can be derived from Eqs. (27)–(29) using Eqs. (40), (43), and (45) as

46 $$\:\stackrel{-}{{\sigma\:}_{xx}}\left(x\right)=2{N}_{1}{w}^{2}m{\text{e}}^{mx}-2{N}_{2}{w}^{2}m{\text{e}}^{-mx}+{\sum\:}_{n=1}^{2}\frac{2{w}^{2}+{\beta\:}^{2}{f}^{2}}{{k}_{n}^{2}-{w}^{2}}\left[{R}_{n}{\:\text{e}}^{{k}_{n}x}+{R}_{n+2}{\:\text{e}}^{-{k}_{n}x}\right],$$

47 $$\:\stackrel{-}{{\sigma\:}_{yy}}\left(x\right)=-2{N}_{1}{w}^{2}m{\text{e}}^{mx}+2{N}_{2}{w}^{2}m{\text{e}}^{-mx}+{\sum\:}_{n=1}^{2}\frac{-2{k}_{n}^{2}+{\beta\:}^{2}{f}^{2}}{{k}_{n}^{2}-{w}^{2}}\left[{R}_{n}{\:\text{e}}^{{k}_{n}x}+{R}_{n+2}{\:\text{e}}^{-{k}_{n}x}\right],$$

48 $$\:\stackrel{-}{{\sigma\:}_{xy}}\left(x\right)=iw\left\{\left({w}^{2}+{m}^{2}\right)\left({N}_{1}{\text{e}}^{mx}+{N}_{2}{\text{e}}^{-mx}\right)+{\sum\:}_{n=1}^{2}\frac{2{k}_{n}}{{k}_{n}^{2}-{w}^{2}}\left[{R}_{n}{\:\text{e}}^{{k}_{n}x}-{R}_{n+2}{\:\text{e}}^{-{k}_{n}x}\right]\right\}.$$

The normal mode analysis is used to find the solution in the Fourier-transformed domain. Assuming that the normal mode analysis for these functions can occur because all of the relations on the real line are smooth enough.

The following equations emerge from plugging the considered variable expressions into the boundary conditions (11)

49 $$\:{\sum\:}_{n=1}^{2}\frac{{k}_{n}^{2}-{w}^{2}-{f}^{2}}{{k}_{n}^{2}-{w}^{2}}[{R}_{n}+{R}_{n+2}]=\stackrel{-}{h},$$

50 $$\:{\sum\:}_{n=1}^{2}\frac{{k}_{n}^{2}-{w}^{2}-{f}^{2}}{{k}_{n}^{2}-{w}^{2}}[{R}_{n}{\:\text{e}}^{{k}_{n}{L}_{x}}+{R}_{n+2}{\:\text{e}}^{-{k}_{n}{L}_{x}}]=0,$$

51 $$\:{\sum\:}_{n=1}^{2}\frac{2{w}^{2}+{\beta\:}^{2}{f}^{2}}{{k}_{n}^{2}-{w}^{2}}\left[{R}_{n}+{R}_{n+2}\right]+2{N}_{1}{w}^{2}m-2{N}_{2}{w}^{2}m=0,$$

52 $$\:{\sum\:}_{n=1}^{2}\frac{2{w}^{2}+{\beta\:}^{2}{f}^{2}}{{k}_{n}^{2}-{w}^{2}}\left[{R}_{n}{\:\text{e}}^{{k}_{n}{L}_{x}}+{R}_{n+2}{\:\text{e}}^{-{k}_{n}{L}_{x}}\right]+2{N}_{1}{w}^{2}m{\text{e}}^{m{L}_{x}}-2{N}_{2}{w}^{2}m{\text{e}}^{-m{L}_{x}}=0,$$

53 $$\:{\sum\:}_{n=1}^{2}\frac{2{k}_{n}}{{k}_{n}^{2}-{w}^{2}}\left[{R}_{n}-{R}_{n+2}\right]+\left({w}^{2}+{m}^{2}\right)\left({N}_{1}+{N}_{2}\right)=0,$$

54 $$\:{\sum\:}_{n=1}^{2}\frac{2{k}_{n}}{{k}_{n}^{2}-{w}^{2}}\left[{R}_{n}{\:\text{e}}^{{k}_{n}{L}_{x}}-{R}_{n+2}{\:\text{e}}^{-{k}_{n}{L}_{x}}\right]+\left({w}^{2}+{m}^{2}\right)\left({N}_{1}{\text{e}}^{m{L}_{x}}+{N}_{2}{\text{e}}^{-m{L}_{x}}\right)=0.$$

It is possible to solve the above linear system and determine the integration constants $\:{R}_{l}\left(l=1,\:2,\:3,\:4\right)$, $\:{N}_{1}$ and $\:{N}_{2}.$

The burn evaluation states that thermal injury occurs when the temperature of the basal layer, which acts as the interface between the epidermis and dermis, rises above 44 °C^45^. The evaluation of thermal damage is essential for living tissue and its therapeutic uses in bioengineering. The first to introduce a numerical approach for evaluating thermal damage was Moritz and Henriques^46^. In this assessment, the damage parameter is determined using the Arrenius equation^47^. The damage rate can be determined by

55 $$\:\mathcal{D}={F}_{f}\underset{0}{\overset{t}{\int\:}}{e}^{-\frac{{E}_{a}}{RT}}d\mathcal{t}$$

where $\:R$ is the universal gas constant, $\:{E}_{a}$ is the energy of activation for the denaturation reaction, and $\:{F}_{f}$ is the frequency factor^48^.

## The computed data and interpretation

This section examines thermal transmission and the relations between mechanical forces and temperature for skin tissue exposed to varying heat loading. The study makes use of the generalized fourth-order MGT thermoelastic model. The way that heat loading is supplied to the skin tissue’s outer surface is interpreted as

56 $$\:h\left(y,\:t\right)={\theta\:}_{0}U\left({t}_{s}-t\right)U\left({L}_{y}-y\right)\text{c}\text{o}\text{s}\left(\frac{\pi\:}{2{t}_{s}}t\right),$$

where $\:{t}_{s}$ is the thermal loading exposure duration, $\:{\theta\:}_{0}>0$ is the thermal loading strength, and $\:U\left(.\right)$ is the unit step function.

The following numerical computations and comparisons will make use of the biological and thermal properties of skin tissue shown in Table 1^39,48^.

**Table 1:** Thermophysical parameters of skin tissue.

Parameter	Value	Parameter	Value
$\:{\lambda\:}_{t}$	$\:8.27\times\:1{0}^{8}\:\text{k}\text{g}/\left(\text{m}\:{\text{s}}^{2}\right)$	$\:{\mu\:}_{t}$	$\:3.446\times\:1{0}^{7}\:\text{k}\text{g}/\left(\text{m}\:{\text{s}}^{2}\right)$
$\:{\alpha\:}_{t}$	$\:1\times\:1{0}^{-4}\:1/\text{K}$	$\:{\mathcal{K}}_{t}$	$\:0.628\:\text{J}/\left(\text{s}\:\text{m}\:\text{K}\right)$
$\:{\mathcal{K}}_{t}^{*}$	$\:0.0628\:\text{J}/\left({\text{s}}^{2}\:\text{m}\:\text{K}\right)$	$\:{c}_{t}$	$\:4187\:\text{J}/\left(\text{k}\text{g}\:\text{K}\right)$
$\:{S}_{im}$	$\:1.19\times\:1{0}^{3}\:\text{W}/{\text{m}}^{3}$	$\:{c}_{m}$	$\:0.1\:1/\text{K}$
$\:{\rho\:}_{b}$	$\:1060\:\text{k}\text{g}/{\text{m}}^{3}$	$\:{c}_{b}$	$\:3860\:\text{J}/\left(\text{k}\text{g}\:K\right)$
$\:{w}_{b}$	$\:0.00187\:1/\text{s}$	$\:{\theta\:}_{0}$	$\:5\:\text{K}$
$\:{F}_{f}$	$\:3.1\times\:1{0}^{98}\:1/\text{s}$	$\:{E}_{a}$	$\:6.28\times\:1{0}^{5}\:\text{J}/\text{m}\text{o}\text{l}$
$\:R$	$\:8.313\:\text{J}/\left(\text{m}\text{o}\text{l}\:\text{K}\right)$	$\:{\rho\:}_{t}$	$\:1000\:\text{k}\text{g}/{\text{m}}^{3}$
$\:{L}_{x}$	$\:0.03\:\text{m}$	$\:{L}_{y}$	$\:0.03\:\text{m}$
$\:{T}_{b}$	$\:310\:\text{K}$	$\:w$	$\:10\:1/\text{m}$
$\:f$	$\:15\:1/\text{s}$	$\:{\tau\:}_{h}$	$\:8\:\text{s}$

**Fig. 2:**
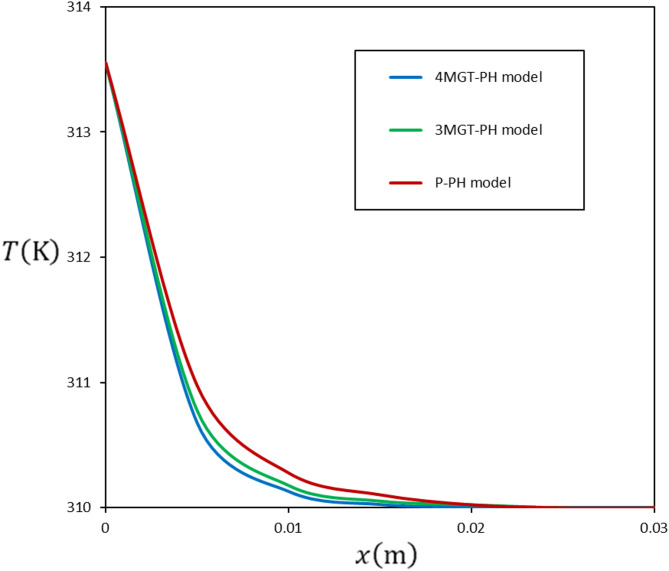
Record of temperature $\:T$ over the distance $\:x$ for various models.

**Fig. 3:**
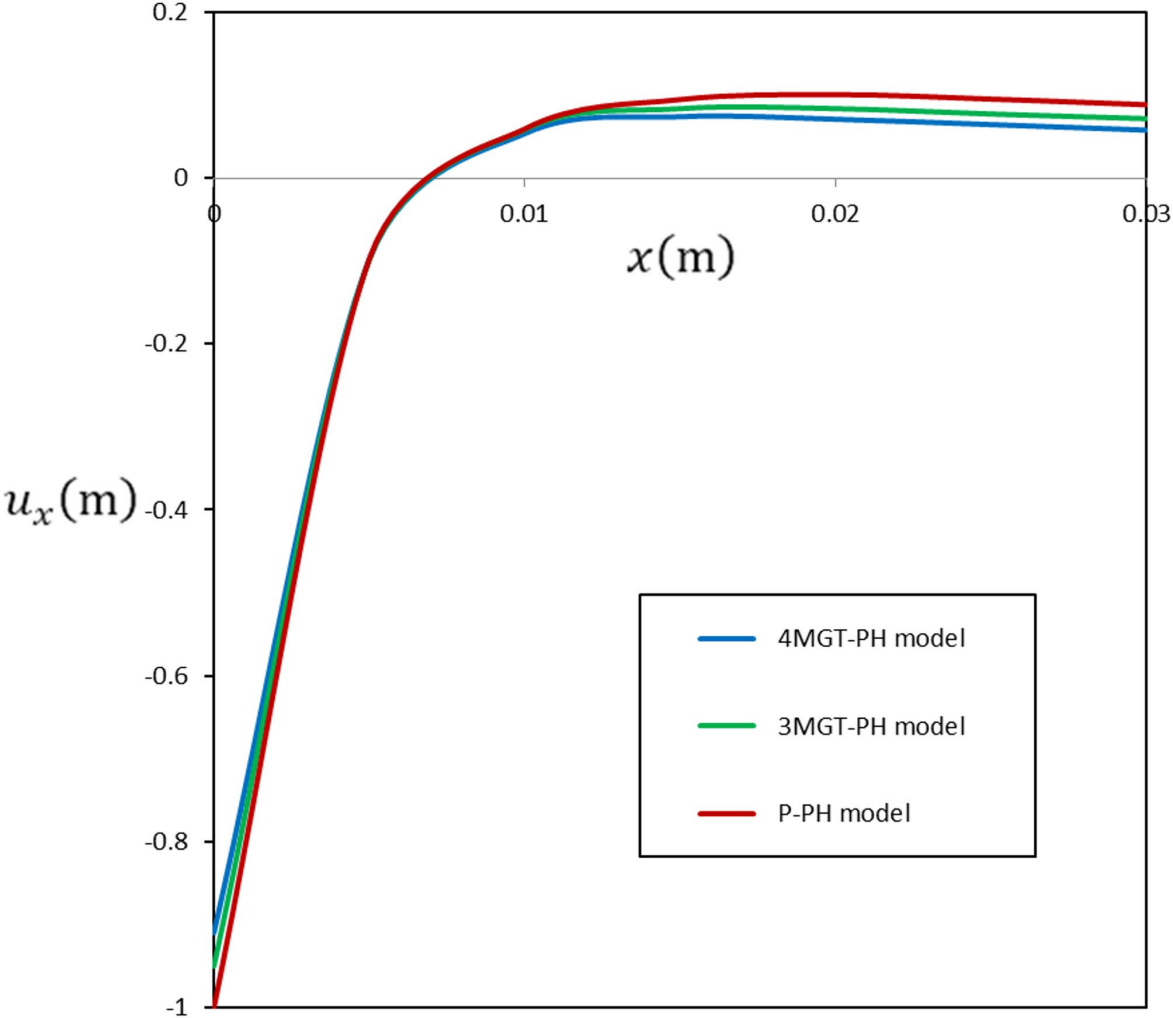
Record of displacement $\:{u}_{x}$ over the distance $\:x$ for various models.

**Fig. 4:**
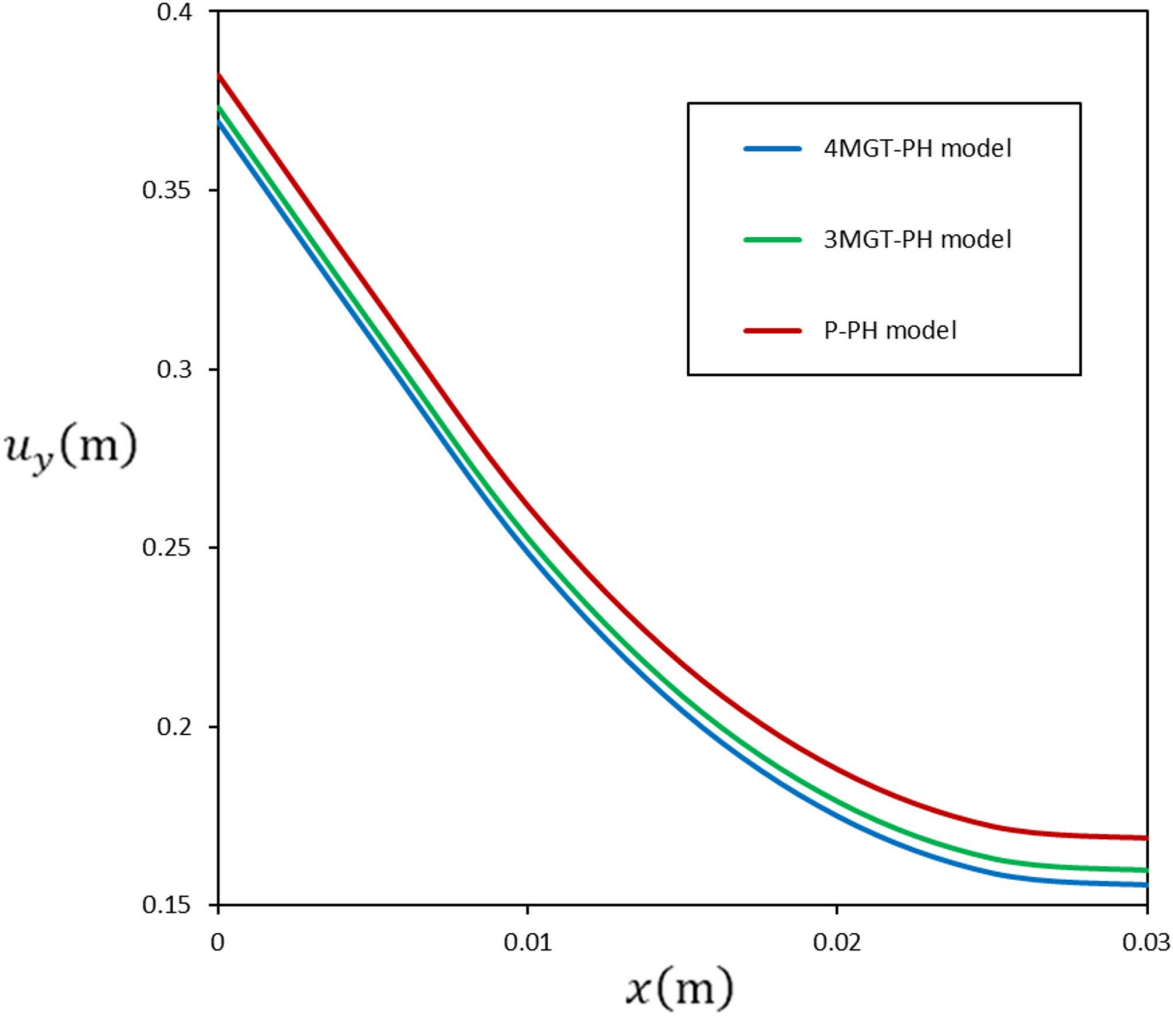
Record of displacement $\:{u}_{y}$ over the distance $\:x$ for various models.

**Fig. 5:**
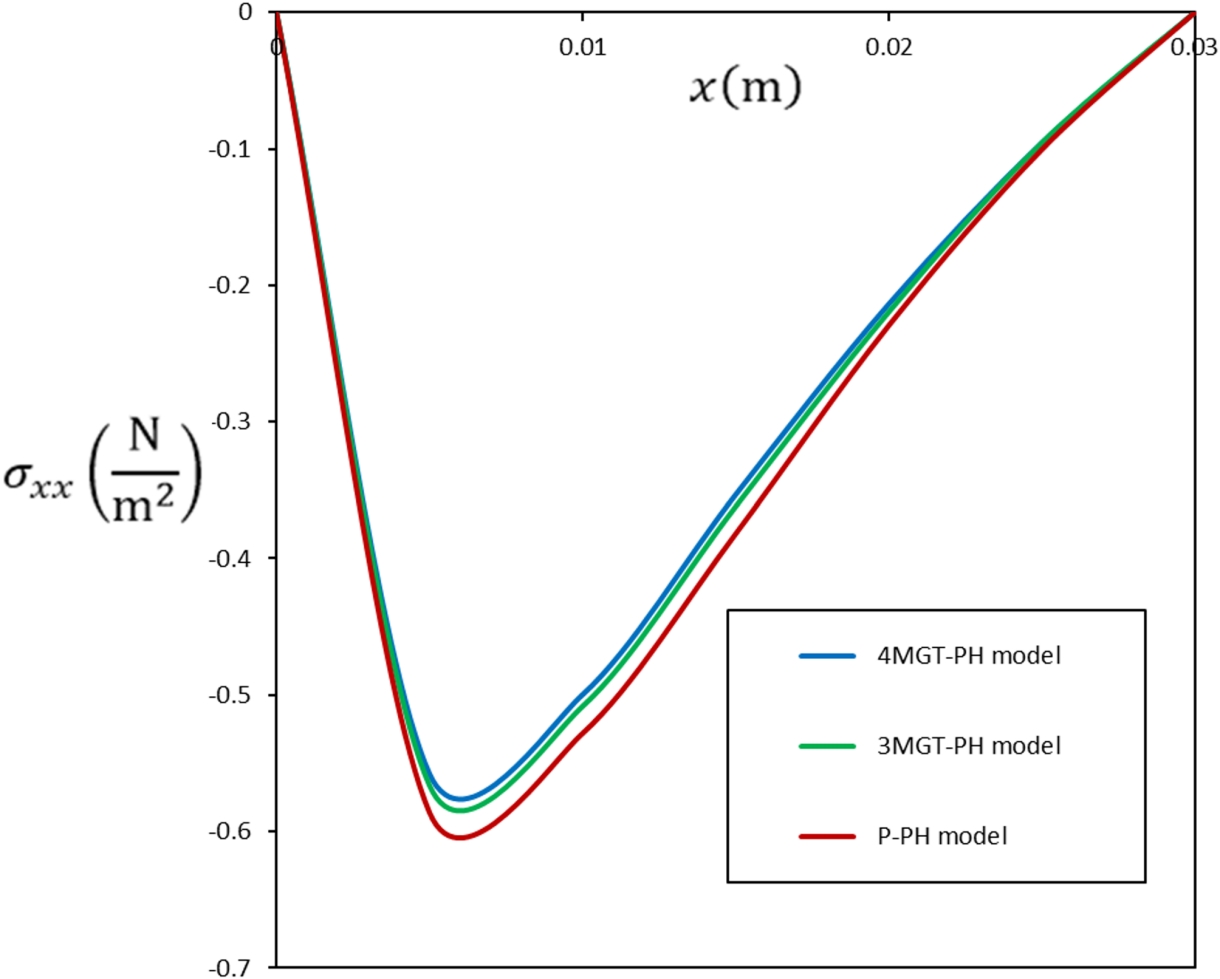
Record of stress $\:{\sigma\:}_{xx}$ over the distance $\:x$ for various models.

**Fig. 6:**
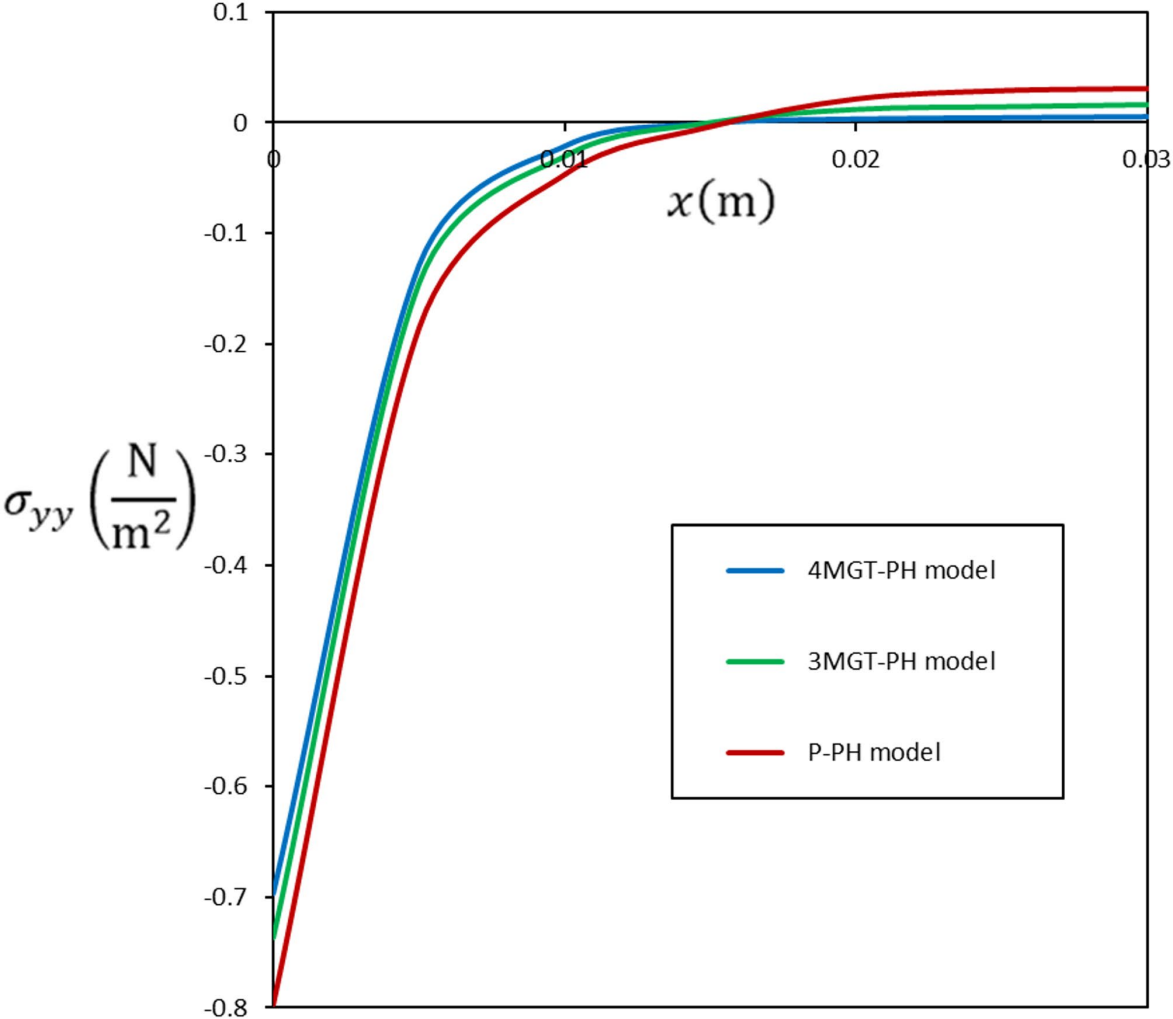
Record of stress $\:{\sigma\:}_{yy}$ over the distance $\:x$ for various models.

**Fig. 7:**
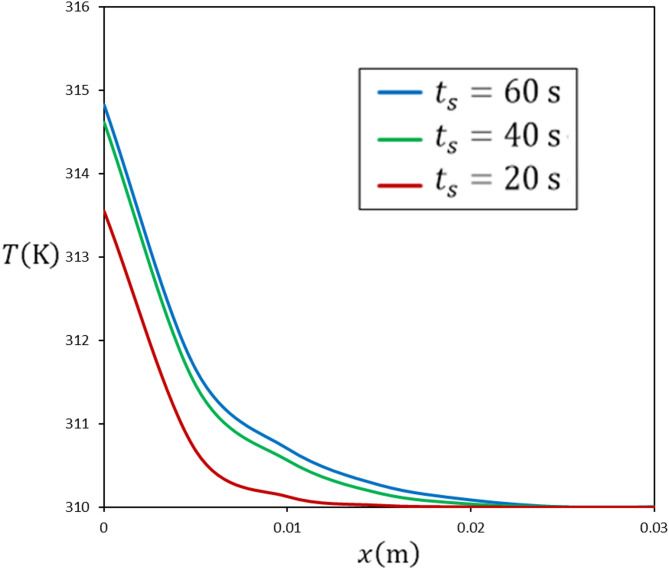
The impact of the heat loading exposure duration $\:{t}_{s}$ over temperature $\:T$.

**Fig. 8:**
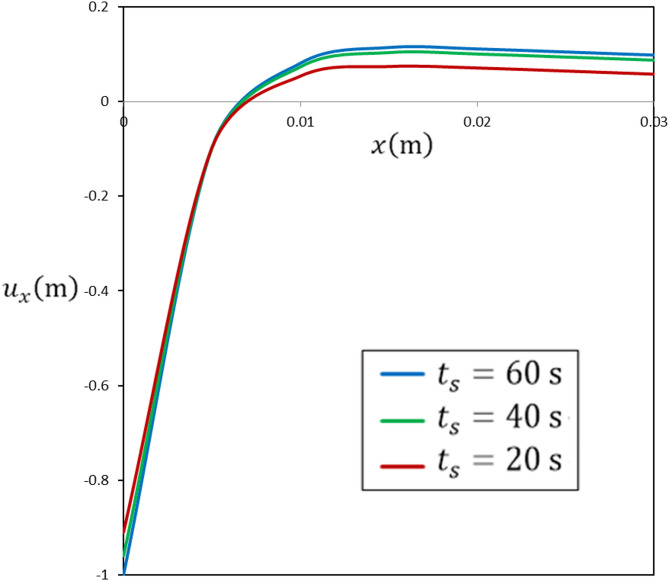
The impact of the heat loading exposure duration $\:{t}_{s}$ over displacement $\:{u}_{x}$.

**Fig. 9:**
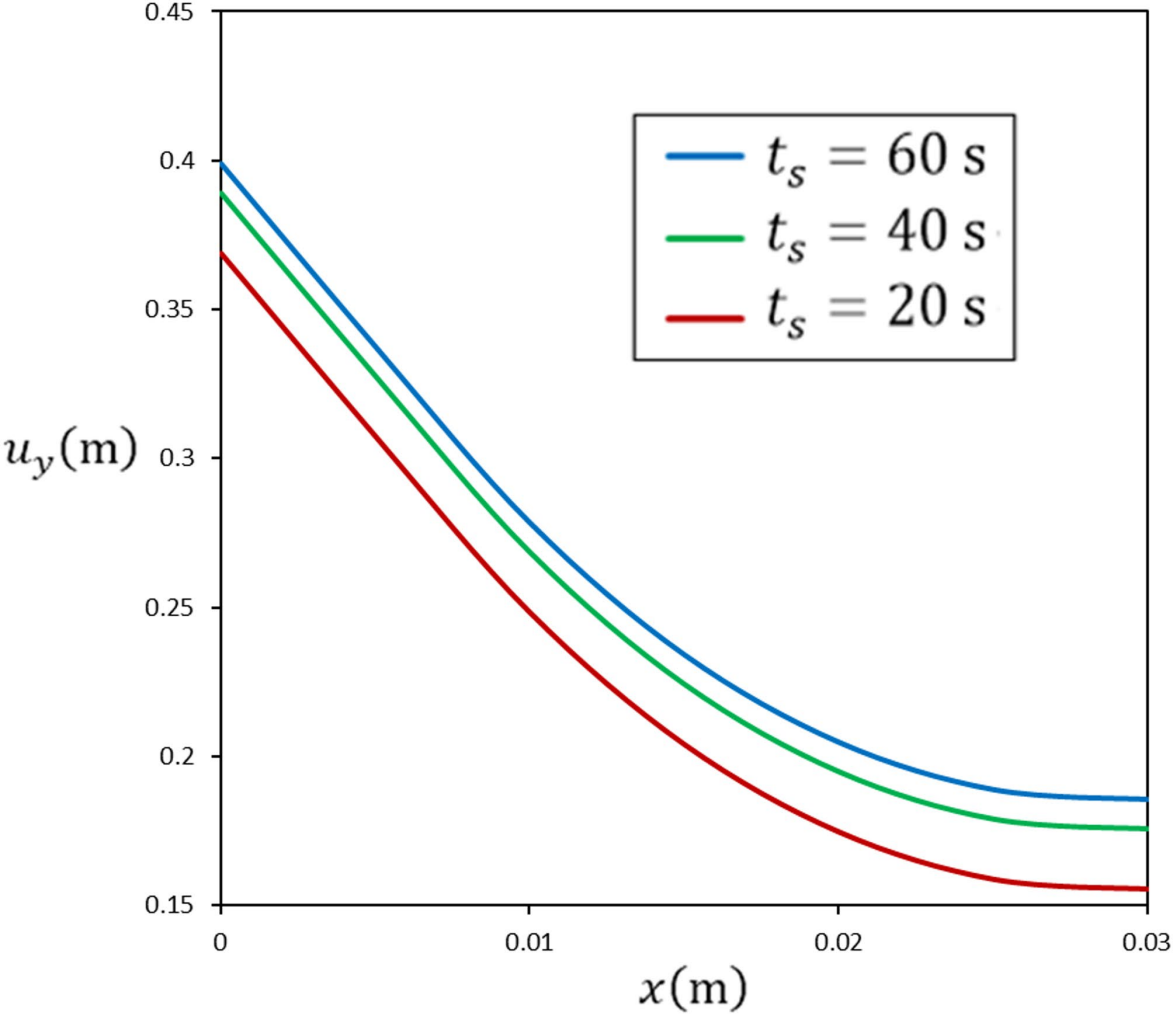
The impact of the heat loading exposure duration $\:{t}_{s}$ over displacement $\:{u}_{y}$.

**Fig. 10:**
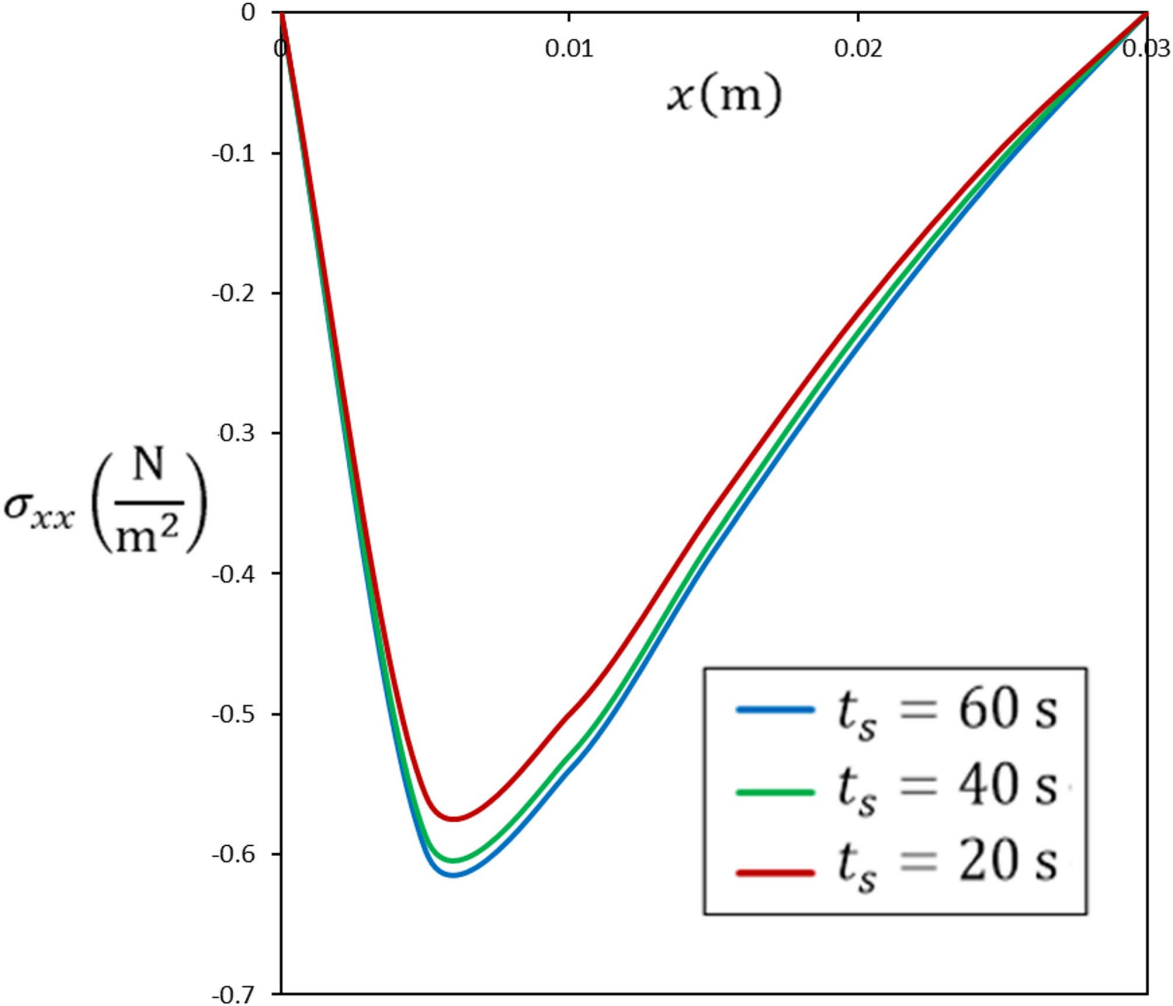
The impact of the heat loading exposure duration $\:{t}_{s}$ over stress $\:{\sigma\:}_{xx}$.

**Fig. 11:**
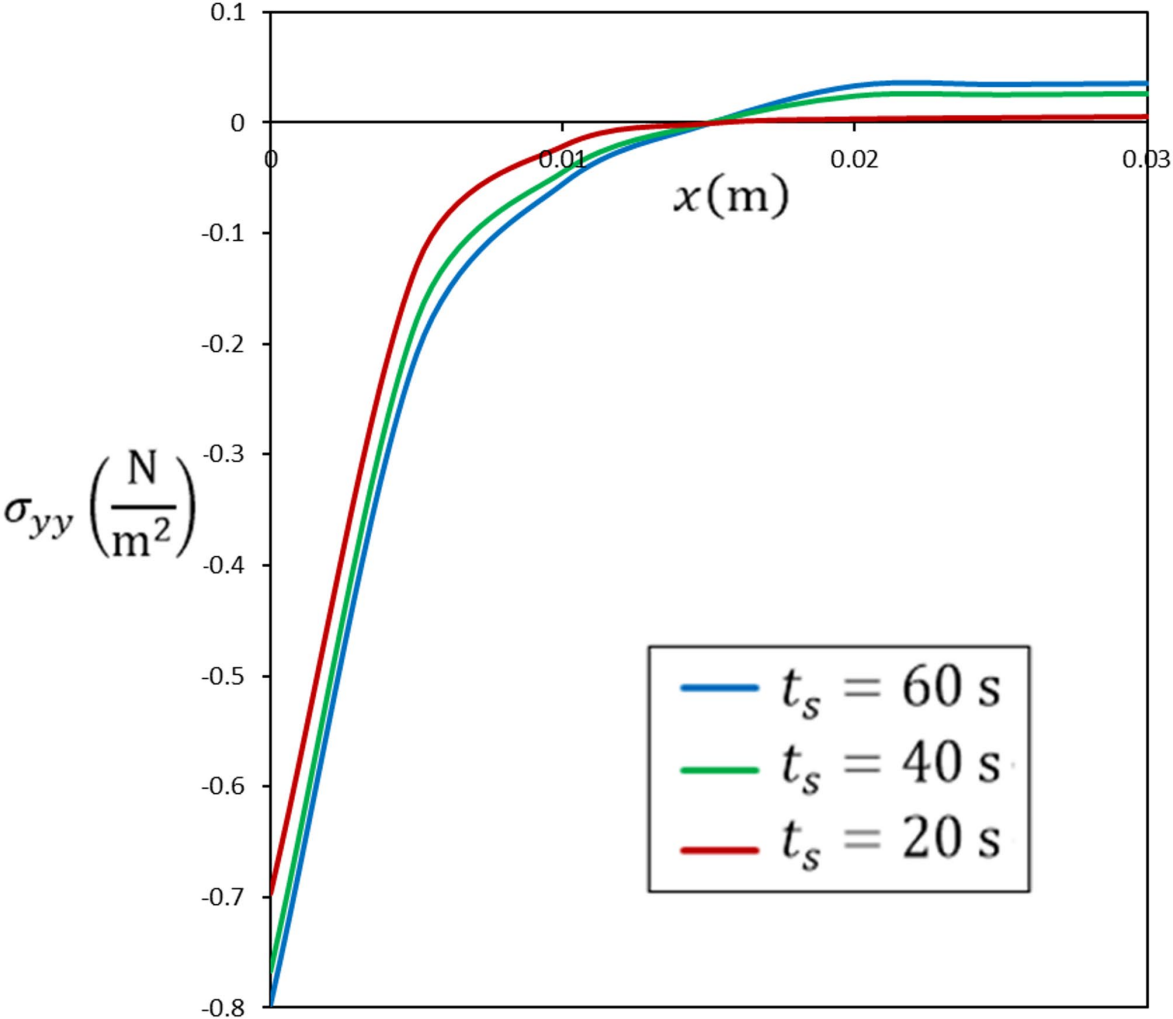
The impact of the heat loading exposure duration $\:{t}_{s}$ over stress $\:{\sigma\:}_{yy}$.

**Fig. 12:**
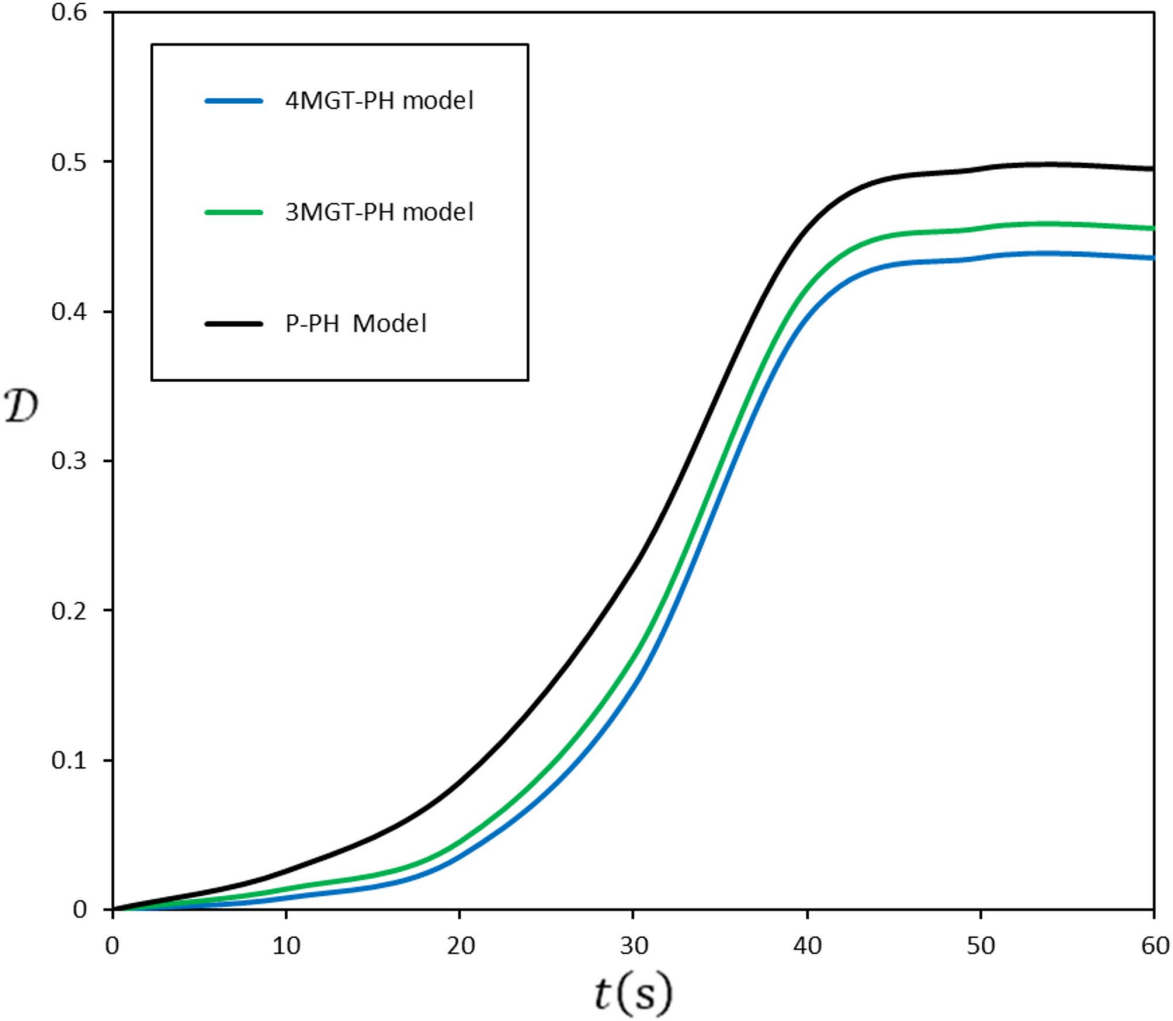
Comparison between the models for the evolution of thermal damage $\:\mathcal{D}$.

Figures 2, 3, 4, 5, 6, 7, 8, 9, 10, 11 and 12 display the numerical outcomes for different locations throughout the $\:x$-axis and the thermal damage along the $\:t$-time. The study will assess how specific influential parameters, such as relaxation (delay) time, model order, and duration of heat loading exposure, interact with skin tissues. The figures’ meticulous examination, sensitivities of parameters, and linkages to prior research will offer a thorough assessment of the fourth-order MGT thermoelastic model and its capacity to faithfully depict skin tissue’s mechanical and thermal reactions exposed to the varying thermal loading.

The primary objective of improving bio-thermal models is to resolve the intrinsic conflict in the Pennes model for bio-thermal transmission, which makes the assumption that thermal waves move at infinite rates. Therefore, a comparison of bioheat thermoelastic models will be covered in this initial scenario. The fourth-order Moore–Gibson–Thompson biothermal transfer model (4MGT-PH), the third-order Moore–Gibson–Thompson bio-thermal transmission model (3MGT-PH), and the conventional Pennes bio-thermal transmission parabolic model (P-PH) are the three different biothermal models used in this study to compare the locations of different physical variables inside skin tissues.

The physical field variables that were investigated at different distances $\:x$ across several thermal models are shown in Figs. 1, 2, 3, 4 and 5. The parameters $\:y=0.03\:\text{m}$, $\:\:{t}_{s}=20\:\text{s}$, and $\:t=10\:\text{s}$are employed in numerical computations. The findings show a distinct difference between the 4MGT-PH, 3MGT-PH, and P-PH models’ outputs. The various field distributions are significantly influenced by the model’s order as well as the thermal coefficient $\:{\tau\:}_{h}.$

Figure 2 shows the thermodynamic temperature $\:T$ changes as a function of distance $\:x$ for the three thermoelasticity models. The graph shows that the P-PH model has higher temperature values than the 4MGT-PH and 3MGT-PH models. Interestingly, heat waves move more slowly in the 4MGT-PH and 3MGT-PH models than in the P-PH version. This discrepancy is caused by the thermal relaxation periods ($\:{\tau\:}_{h}$) that are present in both updated models. This finding further supports the accuracy of the results generated by the suggested model, which offers a more accurate representation of thermal waves propagating through skin tissues.

In addition to the previously reported results, the 4MGT-PH model outperforms the P-PH and 3MGT-PH models in terms of prediction abilities because of incorporating additional higher-order terms. The enhanced efficiency of the 4MGT-PH model is ascribed to its capability to disperse heat more quickly and efficiently. Additionally, the behavior of the 3MGT-PH and 4MGT-PH models is rather similar. This result is in line with previous research^49,50^, which demonstrates that the inclusion of a relaxation period lowers the thermal wave properties characteristics and produces behaviors akin to Fourier diffusion.

The issue of thermal waves is resolved by generalized models, which correctly predict that thermal signals move at bounded speeds. When approximating heat conduction in temperature fields that change dynamically, these models are especially helpful. The suggested MGT generalized thermoelasticity model overcomes the heat wave problem by offering a more realistic representation of thermal conductivity and guaranteeing the precision of the numerical results.

The displacements ($\:{u}_{x},\:{u}_{y}$) and stresses ($\:{\sigma\:}_{xx},\:{\sigma\:}_{yy}$) change with distance ($\:x$), as shown in Figs. 3, 4, 5 and 6. The skin tissue reacts to temperature changes more slowly when thermal relaxation periods ($\:{\tau\:}_{h}$) are introduced. The time evolution of the skin tissue’s stress and deformation is significantly impacted by this delay. Compared to traditional models, the MGT models’ displacements ($\:{u}_{x},\:{u}_{y}$) show a delayed heat response, leading to smoother and more dispersed deformation. Furthermore, the MGT models reveal fewer steep gradients in the thermal stress ($\:{\sigma\:}_{xx},\:{\sigma\:}_{yy}$).

The fourth-order model provides additional enhancements by adding more higher-order terms, as can be seen when comparing the third-order and fourth-order MGT models. This development results in more accurate stress and deformation predictions. A more progressive thermal and mechanical response is also shown by the smoother distributions of all physical variables produced by the fourth-order MGT model. The study’s findings support previous research^51–53^, indicating the precision and dependability of the numerical method used.

The importance of the duration of heat loading exposure in skin tissues is covered in the second scenario. The thermal loading exposure period ($\:{t}_{s}$) plays a major role in determining the rate and velocity at which heat is injected into the skin tissue. Both the transient heat transmission process and the thermomechanical reaction of the skin tissue are directly impacted by this characteristic. By looking at numerical data related to significant variables like temperature, displacement, and stress, one can gain a comprehensive understanding of how rapid heating affects skin tissue.

Figures 7, 8, 9, 10 and 11 show how $\:{t}_{s}$ affects the temperature profile ($\:T$), displacements ($\:{u}_{x},\:{u}_{y}$), and thermal stress components ($\:{\sigma\:}_{xx}$, $\:{\sigma\:}_{yy}$). To investigate the effects of $\:{t}_{s}$, numerical simulations were performed for three different thermal loading exposure lengths ($\:{t}_{s}=$20 s, 40 s, and 60 s). The parameters $\:y=0.01$ m and $\:t=10$ s are used simultaneously in numerical computations. The discussion that follows provides a detailed analysis of the results.

The temperature profile investigation indicates that a more pronounced and localized temperature increase inside the skin tissue occurs with a longer thermal loading exposure length ($\:{t}_{s}$) (see Fig. 7). Increased temperature gradients and peak temperatures result in a sharp thermal response. Conversely, a shorter $\:{t}_{s}$ promotes a gradual and consistent increase in temperature, lowering peak temperatures and producing smoother gradients over the skin tissue.

A longer $\:{t}_{s}$, which is linked to a higher rate of heat deposition, physically produces faster thermal responses. The concentrated heating creates steeper thermal gradients, increasing the likelihood of temperature overshoots and short-term variations. Conversely, a shorter $\:{t}_{s}$ allows the temperature to spread more evenly across the skin tissue and reduces the rate of heat deposition. This lowers peak temperatures and prolongs the thermal impacts of the skin tissue.

Longer $\:{t}_{s}$ results in concentrated and rapid thermal expansions and contractions, which create abrupt displacement shifts when examining displacements ($\:{u}_{x},\:{u}_{y}$), as seen in Figs. 8 and 9. Conversely, a shorter $\:{t}_{s}$ results in displacement fields that are more uniform and smooth, with slower transient deformations that progressively get closer to equilibrium. According to the scientific understanding of this phenomena, rapid temperature changes together with a longer $\:{t}_{s}$ amplifying thermal expansion rates lead to significant transient deformations concentrated near the heat source. Conversely, a slower temperature rise is indicated by a shorter $\:{t}_{s}$, which slows down the rate of thermal expansion and produces smoother displacement reactions and more gradual deformation.

Figures 10 and 11 demonstrate that as $\:{t}_{s}$ increases, transient stresses ($\:{\sigma\:}_{xx}$, $\:{\sigma\:}_{yy}$) increase due to rapid temperature gradients and uneven expansion. A shorter $\:{t}_{s}$ efficiently reduces the intensity of stresses ($\:{\sigma\:}_{xx}$, $\:{\sigma\:}_{yy}$), leading to more gradual stress response and smaller stress gradients.

The behaviors of thermomechanical coupling and heat transfer dynamics exhibit notable variations under different thermal loading exposure durations ($\:{t}_{s}$). This variation highlights how skin tissue reactions rely on the duration of thermal loading exposure by demonstrating that shorter durations encourage a more stable and consistent thermal response, whereas longer durations may result in more rapid alterations. Understanding these differences is crucial because they directly affect performance in real-world situations. By varying the duration of heat loading exposure, physicians and researchers can improve the performance skin tissues in a range of applications, including thermal therapy in biological tissues.

The extent of heat damage is covered in the final scenario. Extended exposure to extremely high temperatures can harm the cellular and structural components of the skin by thermally damaging the epidermal tissues. Thermal injury can occur via blistering from hot liquids, connection to heated bodies, fire damage, and even medical operations. The degree of thermal damage is dependent on the duration and intensity of temperature exposure, as well as the inherent susceptibility of the individual.

The assessment of skin tissue burns is essential in the field of medicine. Current studies have shown that heat damage starts when the temperature of the base stratum reaches 44 °C and increases by 0.53 °C at that point^54–56^. The thermal damage to skin tissue following exposure to a heating load for $\:{t}_{s}=$20 seconds at $\:x=0.01$ m and $\:y=0.01$ m is calculated in this work using the composite trapezoidal rule. The findings in Fig. 12 clearly demonstrate a statistically significant variation in the anticipated burn times between the bio-thermal models. Specifically, when applied to varying heating loads, the 4MGT-PH model exhibits lower burn periods than the P-PH model. Consequently, when the Pennes model is unable to adequately capture thermal transmission dynamics, the 4MGT-PH and 3MGT-PH models provide more precise forecasts in high power and rapid heating scenarios.

## Conclusion

By introducing a novel Moore–Gibson–Thomson (MGT) thermoelasticity model, this study sought to advance the subject of thermoelasticity. This novel model provides a more thorough framework for studying thermoelastic events by combining the Moore–Gibson–Thomson equation with the relaxation period of the heat flux vector and higher-order derivatives. The thermo-elastic transient response of two-dimensional skin tissues subjected to varying thermal loads was examined, with thermophysical fields graphically displayed, in order to validate the model. The study examined thermal damage, the effect of the duration of heat loading exposure on the behavior of thermophysical fields, and a detailed comparison of classical and non-classical thermoelasticity models.

The following were some of the key findings:

- The inclusion of relaxation times in the heat conduction equation significantly improves the convergence in solutions for the 4MGT-PH and 3MGT-PH models. The accuracy of the model has improved by better capturing the non-local effects related to thermal and mechanical processes.
- Higher-order derivatives and relaxation periods reduce the propagation of mechanical and thermal waves within biological tissues, according to experimental data. The complex interaction between heat dissipation and mechanical responsiveness over time is suggested by this attenuation.
- Compared to temperature profiles produced by other models, the 4MGT-PH model exhibits a more pronounced and rapid decline in temperature as one moves farther away from the heat source. This implies that the 4MGT-PH model more fully and accurately captures the underlying thermal phenomena within the skin tissue.
- More precisely, a longer heat loading exposure time leads to a faster and more concentrated temperature increase, which in turn produces higher peak temperatures, more pronounced temperature gradients, and stronger transitory effects. Conversely, a shorter heat loading exposure time results in slower, smoother thermal responses, which lessen excessive thermal and mechanical fluctuations.
- The Pennes thermal transmission model (P-PH) and the 3MGT-PH model produce considerably different estimates of temperature and thermal damage than the current bioheat model (4MGT-PH).
- The duration of the burn differs for each of the three heat transfer models when skin tissue is exposed to varying heating loads.
- This modeling technique may eventually improve the precision of heat modeling in biological fields, such as tissue diagnosis and the development of heat therapeutics, by better accounting for the intricate and dynamic structure of thermal transmission in skin tissue.

## Data Availability

All available data are present in the manuscript. All data and models generated or used during the study appear in the submitted article.
